# The Raybrook Hospital for Incipient Consumptives

**Published:** 1907-04

**Authors:** M. P. Burnham, Elmer E. Larkin, Martin E. McClary, J. Bancroft Devins, Edward R. Rice, John H. Pryor

**Affiliations:** Superintendent; Raybrook, N.Y.; Board of Trustees; Raybrook, N.Y.; Board of Trustees; Raybrook, N.Y.; Board of Trustees; Raybrook, N.Y.; Board of Trustees; Raybrook, N.Y.; Board of Trustees; Raybrook, N.Y.


### CORRESPONDENCE

### The Raybrook Hospital for Incipient Consumptives.

**The New York State Hospital for Incipient Tuberculosis, located at Raybrook in the Adirondacks, finds difficulty in securing suitable cases to fill the beds established. The superintendent explains the situation in the letter which follows.**

*To the Medical Profession of New York State:*

At the New York State Hospital for Incipient Tuberculosis there are 35 vacant beds. This is due to the fact that suitable cases for treatment, and for which the hospital was built, cannot be secured. Out of 940 applications for admission to the hospital last year 220 patients were, following examination, received for treatment, the remaining 720 being too far advanced to be accepted. Of the 220 received 65% only were actually incipient cases. The balance, on admission, were found to be suffering from more advanced disease and were re-classified at the hospital. This condition, considering the well known curability of the disease in question in its early stages, should not exist in New York State today, where annually 14,000 people die of tuberculosis. There are approximately 50,000 consumptives in the state. Thousands of these must be in the incipient stage, losing all chance of recovery.

One of the chief purposes of the New York State Hospital is educational, it being designed to encourage early diagnosis and to supply the only ideal form of prevention while saving life. It would seem that incipient tuberculosis is an unknown, unrecognised disease, except by a few experts.

In the last annual report of this hospital it is shown that 85% of incipient cases were discharged apparently recovered. This class constituted 65% of all cases under treatment. Of the moderately advanced cases 23% were discharged apparently recovered and of the advanced cases no per cent. were so discharged. The possibility of obtaining good results is plainly seen, therefore, to depend in the main upon the amount of tuberculosis present when the case is placed under treatment.

The definition of an incipient case, as adopted by the National Society for the Study and Prevention of Tuberculosis, is as follows:

*"Slight initial lesion in the form of infiltration limited to the apex or a small part of one lobe. No tuberculous complications. Slight or no constitutional symptoms (particularly including gastric or intestinal disturbance or rapid loss of weight). Slight or no elevation of temperature or acceleration of pulse at any time during the twenty-four hours, especially after rest. Expectoration usually small in amount or absent. Tubercle bacilli may be present or absent."*

This is the class of cases we desire to receive to the full working capacity of the institution.

The Board of Trustees of the New York State Hospital for Incipient Tuberculosis have authorised the publication of this letter, feeling it their duty to acquaint the medical profession with the surprising conditions existing in the State at this time regarding tuberculosis and its prevention and treatment.

Signed: M. P. BURNHAM, M.D., Superintendent.

Board of Trustees—
ELMER E. LARKIN, M.D.
MARTIN E. MCCLARY.
REV. J. BANCROFT DEVINS, D.D.
EDWARD R. RICE.
JOHN H. PRYOR, M.D.

RAYBROOK, N. Y., March 13, 1907.

